# Implementing Outpatient Therapeutic Playgroups for NICU Families: A Quality Improvement Project

**DOI:** 10.3390/bs16040600

**Published:** 2026-04-17

**Authors:** Mariana C. Aokalani, Katherine L. Wisner, Nickie N. Andescavage, Catherine Limperopoulos, Barbara K. Stuart

**Affiliations:** 1 Children’s National Hospital, 111 Michigan Ave., NW, Washington, DC 20010, USA; kwisner@childrensnational.org (K.L.W.); nniforat@childrensnational.org (N.N.A.); 2 School of Medicine and Health Sciences, George Washington University, Ross Hall, 2300 Eye Street, NW, Washington, DC 20037, USA; 3 Zuckerberg San Francisco General Hospital & Trauma Center, 1001 Potrero Avenue, Building 5, 6B, San Francisco, CA 94110, USA

**Keywords:** implementation science, CFIR, NICU, therapeutic playgroups, mental health services, dyadic intervention

## Abstract

Therapeutic playgroups have shown promise in enhancing caregiver–infant mental health outcomes, yet tailored approaches for families following neonatal intensive care unit (NICU) admission remain limited. In this brief report on Quality Improvement, we evaluate key strategies and challenges in implementing an adapted therapeutic playgroup intervention designed for caregivers and infants with a history of NICU hospitalization at University of California, San Francisco and Zuckerberg San Francisco (UCSF) Zuckerberg San Francisco General Hospital (ZSFG) We conducted semi-structured interviews with NICU psychologists to assess local feasibility, barriers, and facilitators to implementation. Implementation science frameworks—the Consolidated Framework for Implementation Research (CFIR) and Proctor et al.’s implementation outcomes framework (acceptability, adoption, appropriateness, feasibility, and sustainability)—were used to guide data organization and interpretation. Qualitative reporting guidelines were followed to enhance transparency in describing interviews and analytic procedures. The psychologists emphasized the importance of embedding therapeutic playgroups within existing clinical workflows, providing flexible delivery models, and customizing curricula to meet cultural and family-specific needs. Multidisciplinary collaboration enhanced feasibility and parent engagement. Barriers included organizational constraints and variability in caregiver readiness. These findings inform local program development and highlight considerations for integrating dyadic mental health support into post-NICU care. Future work should incorporate caregiver perspectives and explore effective interventions across diverse settings.

## 1. Introduction

In 2023, one in ten infants (9.8%) were admitted to Neonatal Intensive Care Units (NICU) in the United States, representing a 13% increase compared to 2016 NICU admissions of 8.7% (Martin & Osterman, 2025). According to Bradley et al. (2025), between 13 and 15 million infants are born before term gestation worldwide. In 2022, prematurity remained the main cause of death for children under 5 years old globally, accounting for 1 million neonatal deaths.

The multilevel impact of Neonatal Intensive Care Unit (NICU) hospitalization on family mental health, relational dynamics, and early development can have long-lasting adverse effects. Parents of infants in the NICU exhibit substantially higher rates of psychiatric disorders, including perinatal mood and anxiety disorders and post-traumatic stress symptoms (Feldman, 2017; Hoge et al., 2021; Johnson Rolfes & Paulsen, 2022). Infants hospitalized in the NICU are at elevated risk for later psychiatric conditions (e.g., depression, personality disorders) (Feldman, 2016; Grunberg et al., 2020; Shaw et al., 2023), developmental delays, neurodevelopmental disorders such as attention deficit/hyperactivity disorder (ADHD) and autism spectrum disorder (ASD) (Mendelson et al., 2017), and attachment insecurity (Ettenberger et al., 2021).

NICU hospitalization often entails repeated relational disruptions due to infant medical needs, limiting infant access to a consistent, nurturing caregiver who buffers toxic stress. Parents’ experiences of their infant’s medical fragility may foster patterns consistent with Vulnerable Child Syndrome (Green & Solnit, 1964), characterized by negative attributions toward the infant, maladaptive parenting behaviors, and long-term detrimental effects on family functioning and child development (Hartzell et al., 2023). According to relational health scaffolds, attachment quality organizes emerging cognitive, socio-emotional, and early personality systems; thus, early relational disruptions common in the NICU can have enduring developmental consequences (Ettenberger et al., 2021). Further, long-term infant outcomes depend on the family’s adjustment to NICU discharge (Shillington & McNeil, 2021). According to Griffith et al. (2022), the transition to home is often marked by a sense of lack of support and resources. Despite these risks, very few interventions are tailored specifically for medically fragile or NICU-hospitalized infants (Beebe et al., 2018). Existing NICU-based dyadic programs remain limited, predominantly inpatient, and narrow in scope, despite evidence of benefits for caregiver mental health, infant outcomes, and relational health.

To our knowledge, no structured, family-centered dyadic intervention has been implemented following NICU discharge. The post-NICU period is a critical time for parents and infants. Parents frequently feel anxious and unprepared to navigate the transition to home (Engel et al., 2024; DiBari & Rouse, 2023). However, mental health services provided after NICU discharge are scarce (Swenson et al., 2025).

Post-NICU hospitalization interventions often involve parent training, home-visiting, developmental support, and dyadic interaction programs delivered by a range of healthcare providers, including nurses, developmental specialists, psychologists, physicians, and community health workers across Europe, North America, and Australia. Examples include programs such as the Mother–Infant Transaction Program (MITP), Visiting Nurse Association (VNA) home-visiting services, the Stockholm Preterm Interaction-Based Intervention (SPIBI), Hospital to Home: Optimizing the Infant’s Environment (H-HOPE), Following Baby Back Home (FBBH), and Victorian Infant Brain Studies (VIBeS) Plus. Many of these programs emphasize interdisciplinary collaboration and nurse-led follow-up care to support parent–infant interaction, developmental monitoring, and caregiver well-being after NICU discharge.

In addition to in-person services, remote communication and digital health approaches have increasingly been used to support families following NICU discharge. These may include scheduled phone or video calls, text-message check-ins, and email-based follow-up questionnaires conducted at specific intervals depending on program protocols. Mobile health applications such as NICU-2-Home, NeoRaksha, Care@Distance, and other family-focused platforms allow parents to track infant health indicators (e.g., growth, developmental milestones, immunizations, and medical appointments) while also providing educational resources and opportunities for social support. These approaches have been associated with improvements in parental engagement, perceived support, and self-efficacy during the transition from hospital to home (Garfield et al., 2016; Gund et al., 2013; Nayak et al., 2019; Hägi-Pedersen et al., 2022). In several countries, nurse-led follow-up programs and parent support groups play a central role in post-NICU care, providing ongoing education, developmental guidance, and emotional support to families during the transition to home. Multi-family therapeutic playgroups may provide support for caregivers and infants following NICU discharge by addressing multiple domains, including caregiver mental health, relational health, and infant development. Group-based interventions have demonstrated similar relational and developmental benefits in lower-risk infant and toddler populations (Bleiberg & Markowitz, 2019; Deans et al., 2014; Melnyk et al., 2006), using flexible, caregiver-focused, culturally responsive approaches (Gross et al., 2021). In these models, caregivers and their infants typically meet weekly in small groups facilitated by trained clinicians. Sessions generally last 90 min when delivered via telehealth and up to 120 min for in-person formats and include a combination of guided caregiver–infant play, psychoeducation, and discussion of parenting challenges. Facilitators integrate evidence-based treatments (e.g., Parent–Child Interaction Therapy; Cognitive Behavioral Therapy–Mindfulness) with psychoeducational components alongside developmentally appropriate activities such as sensorial or exploratory play (Gross et al., 2021; Antenucci et al., 2024). Therapeutic playgroups can be feasibly delivered through videoconferencing platforms with good caregiver engagement and acceptability (Gross et al., 2021). Early interventions that strengthen maternal sensitivity, parental attributions, and caregiver self-efficacy have been associated with improved parent–infant relationship quality and greater attachment security (Cerqueira & Vozar, 2023). This Quality Improvement initiative was conducted at UCSF/Zuckerberg San Francisco General Hospital to assess the local implementation and feasibility of a therapeutic playgroup model—I See You! Play (Aokalani, 2024). It offers a roadmap for implementing an adapted therapeutic playgroup designed for infants with a history of NICU hospitalization and their caregivers. We explored implementation considerations, including evaluation of existing care protocols, unmet needs, and gaps in post-NICU discharge services.

## 2. Methods

### 2.1. Study Design

This capstone Quality Improvement (QI) project used semi-structured interviews to inform local implementation planning. Interviews were conducted in person or via virtual videoconferencing. The Consolidated Criteria for Reporting Qualitative Research (COREQ) checklist (Tong et al., 2007) was used to guide transparent reporting of interview procedures and analytic processes.

### 2.2. Eligibility

Inclusion criteria included psychologists who had worked for at least one year in a level IV academic NICU at UCSF/Zuckerberg San Francisco General Hospital (ZSFG) (San Francisco, CA, USA), and who were still affiliated with UCSF (San Francisco, CA, USA). Staff with less than one year of experience working with NICU families. Volunteers did not have to be actively providing behavioral health services in the ZSFG NICU (San Francisco, CA, USA).

### 2.3. Instrument

An interview guide was developed to explore five key outcomes described in the Implementation Outcomes Framework (Proctor et al., 2011): (1) Acceptability (i.e., extent to which intervention is satisfactory, agreeable, or implementable); (2) Adoption (i.e., initial uptake or intention to use an intervention); (3) Appropriateness (i.e., perceived fit, relevance, or compatibility of the intervention); (4) Feasibility (i.e., extent to which intervention can be successfully carried out or integrated); and (5) Sustainability (i.e., degree to which an intervention can be maintained over time). The guide also explored contextual aspects of NICU care, including multidisciplinary team dynamics, organizational structures, and systemic barriers commonly observed in academic medical centers.

For clarity, interview questions were organized into five topical domains: (1) Goodness of Fit; (2) Implementation Barriers (e.g., limited staffing, space constraints); (3) Strategies to Address Common Challenges (e.g., integration with existing programs); (4) Engagement Strategies; and (5) Implementation Next Steps. Each interview lasted approximately 30–45 min and was audio-recorded with the volunteers’ verbal consent.

### 2.4. Data Analysis

Interviews were conducted by a bilingual cisgender female psychology intern at the University of California, San Francisco (UCSF) with experience in perinatal mental health. Interviews were conducted via Zoom and audio-recorded with volunteers’ verbal consent. Zoom’s automated transcription function was used to generate initial transcripts, which were subsequently reviewed for accuracy immediately after the interview. Transcripts were shared with volunteers for confirmation and clarification before analysis. Confirmation was provided within 48 h of the interview. After transcript verification, the video recordings were deleted. Verified transcripts were then reviewed and coded to identify implementation-related themes. To enhance analytic consistency, the coding and interpretation were reviewed by a second analyst, and discrepancies were resolved through discussion. Participants were also invited to provide feedback on the interpretation of findings to ensure that reported insights reflected their perspectives.

Initial coding categories included infant medical needs, caregiver emotional states, systemic challenges, and funding considerations. These categories were used to organize responses related to commonly anticipated implementation considerations. Data was inductively coded for the additional themes that emerged during interviews (e.g., transportation, staffing).

To guide data collection and analysis, we integrated the framework of Proctor et al. (2011) with the Consolidated Framework for Implementation Research (CFIR). Together, these frameworks provided a structured approach to understanding barriers, facilitators, and opportunities for improving intervention implementation in clinical practice. The final set of themes was mapped onto relevant constructs from the CFIR to facilitate the interpretation of implementation-related themes and guide future applications into actionable implementation strategies (see Table 1).

**Figure 1:**
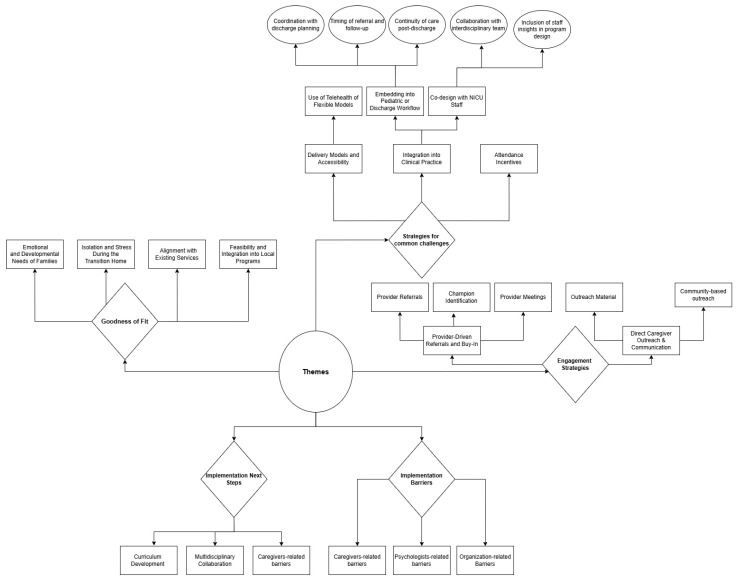
Tree Structure.

All codes were transcribed using Microsoft Excel. Responses were reviewed line-by-line and categorized according to the five outcome domains. A second reviewer examined the coding to ensure consistency, and discrepancies were resolved through discussion. Figure 1 presents a visual representation of the resulting code structure. Themes and sub-themes were then organized into a cross-case matrix in Excel, grouping quotes and insights by participant and domain (see Table 2). This matrix supported systematic comparison across cases, identification of recurring patterns, and the recognition of both convergent and divergent viewpoints. All coding and matrix development were conducted using Microsoft Excel.

## 3. Addressing Bias

To minimize potential bias in data collection and interpretation, several steps were taken. The interview guide and analytic approach were reviewed by two faculty members who were not involved in the intervention development to help mitigate potential bias. Volunteers had no role or vested interest in its implementation. Culturally sensitive interviews were conducted. The interviewer and interviewees had no supervisory or evaluative relationship. Consistency across interviews and accuracy of the interview transcription were maintained. A faculty psychologist overseeing this QI project supported consistency of interpretation by reviewing the analysis. Similarly, participants were invited to provide feedback to ensure that the interpretations reflected their perspectives.

## 4. Ethical Considerations

This project was conducted as a quality improvement initiative as part of an institutional capstone requirement at the University of California, San Francisco. The project was reviewed and supervised by faculty advisors to ensure adherence to ethical standards for quality improvement activities. Because the initiative focused on local program development and implementation planning and did not involve patient data, formal Institutional Review Board review was not required under institutional capstone guidelines.

To ensure participant rights and research ethics were upheld, this quality improvement study was reviewed and approved by faculty advisors affiliated with the university to comply with best practice ethical standards. The participating psychologists were fully informed about the voluntary nature of participation, provided verbal consent before interviews, and were assured of their right to withdraw at any time without penalty. Measures were taken to ensure confidentiality and secure handling of all data.

## 5. Results

### 5.1. Volunteers

All four volunteers were identified as bilingual cisgender Hispanic women. At the time of the interview, two were actively providing mental health services in the NICU. The ages of the psychologists ranged between 28 and 66 years old. One participant was a second-year postdoctoral fellow, two psychologists were faculty members, and the remaining participant was retired and working part-time for UCSF. Their NICU experience ranged from 18 months to 32 years. All UCSF faculty members who met the eligibility criteria were included. The small number of volunteers reflects the limited pool of eligible NICU mental health providers at the study site and the project’s focus on gathering local perspectives to inform local implementation planning.

### 5.2. Key Implementation Finding

Key themes and sub-themes were identified by a structured review of interview responses. A visual coding structure (Figure 1) illustrates the organization of themes and sub-themes identified from the interviews. Findings are summarized in Table 1, including theme descriptions, frequency of responses, and illustrative participant quotes. To further contextualize these findings, themes were mapped onto relevant domains and constructs of the CFIR (see Table 3). This mapping supports the interpretation of implementation barriers and facilitators, and informs this site’s future planning for adopting and sustaining the therapeutic playgroup model.

1. Goodness of Fit

Four key themes emerged. Each corresponds to CFIR constructs: Emotional and developmental needs of families, Isolation and stress during the transition home, Alignment with existing services, and Feasibility and integration into local programs. See Table 3 for detailed information.

Emotional and Developmental Needs of Families: All volunteers indicated that the playgroup model aligns with families’ emotional and developmental needs, reduces isolation and stress. Three psychologists addressed developmental concerns. CFIR: Intervention Characteristics—Relative Advantage.

Isolation and Stress During the Transition Home: All participants emphasized the importance of supporting families during the vulnerable transition home, the need for continued services, and the lack of outpatient mental health infrastructure. CFIR: Outer Setting—Patient Needs & Resources.

Alignment with Existing Services: All psychologists agreed that the model is well-suited for the existing system of care. Three volunteers pointed to this therapeutic playgroup being aligned with institutional current mental health services and fitting within maternal–infant service workflows. CFIR: Inner Setting—Compatibility.

Feasibility and Integration into Local Programs: Three participants noted the feasibility of implementation within local programs, strong alignment with regionally established initiatives, and overall sustainability of integration. CFIR: Inner Setting—Networks & Communication; Outer Setting—Cosmopolitanism.

2. Implementation Barriers

Three primary categories emerged. Each corresponds to CFIR constructs: Caregiver-related barriers, Clinician-related barriers, and Organization-related barriers. See Table 3 for more information.

Caregiver-Related Barriers: Three psychologists described concerns about fear of judgment or inadequacy; interactions with medical staff influenced engagement. One volunteer emphasized the importance of hospital environments, lack of emotional readiness, and stigma around mental health referrals. CFIR: Characteristics of Individuals; Inner Setting; Individual Stage of Change; Outer Setting.

Clinician-Related Barriers: Three participants mentioned competing responsibilities limiting clinicians’ ability to facilitate groups, and one participant noted that variability in organizational readiness can impact program development. CFIR: Inner Setting—Readiness for Implementation.

Organization-Related Barriers: All volunteers highlighted the need for dedicated operational funding. Three psychologists emphasized stronger caregiver engagement mechanisms. Two participants focused on transportation-related obstacles. CFIR: Inner Setting & Outer Setting.

3. Strategies to Address Common Challenges

Four key strategies were identified and organized into multiple CFIR domains. Results will be reviewed in the following themes: “Integration into Clinical Practice,” “Delivery Models and Accessibility,” “Delivery Models and Accessibility,” and “Attendance Incentives.” See Table 3 for additional information.

Integration into Clinical Practice: All volunteers emphasized introducing playgroups during NICU admission or at discharge. Three psychologists referred to embedding information in discharge paperwork, optimizing timing for caregiver participation. Two participants noted involvement of NICU personnel in design/rollout and stakeholder engagement. CFIR: Inner Setting; Outer Setting; Process—Engaging Stakeholders.

Delivery Models and Accessibility: Three volunteers endorsed hybrid or telehealth delivery to improve feasibility and reduce transportation barriers. CFIR: Intervention Characteristics—Adaptability; Outer Setting—Patient Needs & Resources.

Attendance Incentives: One psychologist advocated for incentives such as transport vouchers, snacks, or recognition. CFIR: Outer Setting; Process

4. Engagement Strategies

Two themes were identified and organized into multiple CFIR domains. Our findings will be described in Provider-Driven Referrals and Buy-In, and Direct Caregiver Outreach and Communication. See Table 3 to review findings.

Provider-Driven Referrals and Buy-In: All participants emphasized internal NICU collaboration and referrals. Three volunteers mentioned the importance of expanding referrals to pediatricians and identifying a program champion. CFIR: Inner Setting—Implementation Climate; Process—Champions; Outer Setting—Cosmopolitanism.

Direct Caregiver Outreach and Communication: One psychologist recommended proactive, multimodal outreach and accessible written materials (e.g., flyers, handouts). CFIR: Process—Engaging Volunteers; Intervention Characteristics—Design Quality.

5. Implementation Next Steps

Three key themes emerged—Curriculum Development, Caregiver Readiness and Tailoring, and Multidisciplinary Collaboration. Each corresponds to CFIR constructs. See Table 3 for more information.

Curriculum Development: Two participants endorsed customizing a culturally responsive curriculum and integrating trauma-informed, evidence-based dyadic interventions. CFIR: Intervention Characteristics—Evidence Strength & Quality.

Caregiver Readiness and Tailoring: All psychologists observed high variability in caregiver readiness and infant medical needs, and emphasized tailoring participation to maximize engagement and retention. CFIR: Characteristics of Individuals; Intervention Characteristics—Adaptability.

Multidisciplinary Collaboration: All volunteers recommended embedding the program within existing clinical/community infrastructures, ensuring cross-disciplinary shared responsibility. CFIR: Inner Setting; Outer Setting; Process—Engaging Key Stakeholders.

## 6. Discussion

This brief Quality Improvement report examines implementation considerations for therapeutic playgroups supporting parents and infants post-NICU discharge. Interview insights were organized using CFIR domains (Damschroder et al., 2009) (see Table 3) and Proctor et al.’s (2011) implementation outcomes—acceptability, adoption, appropriateness, feasibility, and sustainability—highlighting key considerations for implementing intervention in a complex safety-net hospital setting.

### 6.1. Key Implementation Insights

Our findings on implementation outcomes align closely with the existing literature on NICU and early relational interventions. Consistent with prior studies (e.g., Baraldi et al., 2020; Griffith et al., 2022; Ghetti et al., 2021), acceptability was highest when programs were culturally responsive, trauma-informed, and delivered flexibly to meet caregiver needs. Similar to H-HOPE and MITPs, adoption relied on provider buy-in and program champions, while alignment with NICU workflows and family priorities supported appropriateness (CFIR: Inner Setting). Feasibility in our study was constrained by resources, echoing previous reports on structural barriers. However, telehealth and hybrid models facilitated participation and broadened reach. This is consistent with recent work emphasizing remote delivery as a solution to transportation and scheduling challenges (McKelvey et al., 2021).

Unlike some traditional interventions that depend heavily on single champions, our findings highlight the role of multilevel stakeholder engagement, cross-sector collaboration, and structured leadership involvement in enhancing sustainability, which aligns with frameworks suggesting that embedding programs into clinical and community systems is essential for long-term impact (Spencer-Smith et al., 2012; Ravn et al., 2012).

Finally, variability in caregiver emotional readiness underscores the need for patient-centered flexibility, adaptive timelines, and individualized outreach. These observations echo recommendations from dyadic and trauma-informed programs (e.g., CPP and TF-CBT), which emphasize matching intervention, timing, and intensity to family needs. Together, these findings suggest that combining evidence-based curricular content with adaptable delivery models and strong stakeholder engagement may maximize effectiveness, feasibility, and acceptability in diverse NICU populations.

At the practice level, integrating therapeutic playgroups into NICU discharge planning and pediatric follow-up pathways may support continuity of relational care as families transition from hospital to community settings. Professionally, interdisciplinary collaboration among psychologists, nurses, developmental specialists, pediatricians, and community providers may strengthen implementation and enhance providers’ capacity to deliver trauma-informed and culturally responsive dyadic support. Future research will be needed to evaluate therapeutic playgroups across diverse healthcare settings and to examine potential impacts on caregiver mental health, parent–infant relationships, and infant developmental trajectories.

While these findings provide important insights into implementation considerations for therapeutic playgroups following NICU discharge, several strengths and limitations of the present quality improvement initiative should be considered when interpreting the results.

### 6.2. Strengths and Limitations

Although this project was conducted as a quality improvement initiative, incorporating elements of the COREQ reporting framework strengthens methodological transparency and allows readers to better understand how data were collected, reviewed, and interpreted. The use of a semi-structured interview guide and secondary review of the analysis further supported consistency in interpretation. Similarly, the use of the Consolidated Framework for Implementation Research (CFIR) to organize implementation-related findings provides a structured approach to identifying barriers and facilitators. Applying an established implementation science framework further provides context to features influencing program delivery and situates the findings within the broader implementation literature. This may better support the transferability of insights to other NICU follow-up programs seeking to implement similar interventions, despite the single-site design.

Several limitations should be considered when interpreting these findings. First, the small sample size may limit the range of perspectives captured and restrict the generalizability of findings to other settings. In addition, this quality improvement initiative was conducted within a single institution (UCSF/Zuckerberg San Francisco General Hospital NICU), which may further limit the applicability of findings to other NICU settings with different organizational structures or resources. Second, interviews were conducted exclusively with psychologists with NICU experiences, which may introduce professional perspective bias and limit the transferability of findings to other healthcare providers involved in post-NICU care, such as nurses, physicians, or developmental specialists. As with many interview-based inquiries, responses may also be subject to recall bias or social desirability bias. Although participants were not directly involved in delivering or planning to implement a therapeutic playgroup at the time of the interview, their familiarity with or interest in such programming may have contributed to positive perceptions. In addition, the interviewer was a clinical psychology intern affiliated with UCSF/ZSFG, and though not embedded within the NICU clinical team, had some degree of professional familiarity with several participants. While the interviewer held no supervisory or evaluative authority over participants, this prior professional relationship may have influenced how participants framed their responses. To mitigate potential response bias, participants were informed that interviews were intended to gather feedback for program improvement, that responses would be de-identified in reporting, and that participation would not affect professional roles or program involvement.

Accordingly, findings should be interpreted as exploratory insights intended to inform local implementation planning and may provide preliminary guidance for similar programs in comparable clinical contexts and program improvement rather than broadly generalizable conclusions.

### 6.3. Future Directions

Although early relational interventions show promising short-term benefits, relatively few studies have examined the impact of therapeutic playgroups for medically fragile infants. Even fewer have examined whether these effects are sustained beyond the immediate post-intervention period. Understanding the short and long-term impact of this type of intervention is particularly important for determining whether improvements in caregiver well-being, infant development, and the parent–infant relationship translate into meaningful and lasting outcomes following NICU discharge. Longitudinal evaluations require additional resources and sustained follow-up; however, assessing outcomes at developmentally relevant timepoints may help determine whether intervention effects persist and inform decisions regarding program adoption and scalability.

Future research may benefit from incorporating multi-level assessment approaches that capture outcomes across caregivers, infants, and the caregiver–infant relationship. At the caregiver level, assessments may include measures of caregivers’ mood and anxiety, parental attributions, and attachment representations. At the infant level, outcomes could include indicators of neurodevelopment and emerging attachment patterns. At the dyadic level, observational assessments of caregiver–infant interaction quality—including maternal sensitivity and the overall quality of the caregiver–infant relationship—may provide important insight into relational change. Integrating these measures within a multidimensional evaluation framework may provide a more comprehensive understanding of intervention impact and help determine whether therapeutic playgroups produce sustained improvements in caregiver functioning, infant development, and early attachment relationships. Such evidence may further guide program refinement and strengthen confidence in implementing therapeutic playgroups as part of NICU follow-up care. To do so, future research should incorporate perspectives from a broader range of NICU stakeholders, including nurses, physicians, developmental specialists, social workers, and caregivers, to ensure that interventions are responsive to the needs and preferences of those directly involved in infant care and family support. Including caregiver perspectives may also provide valuable insight into the acceptability, feasibility, and perceived impact of post-NICU support programs during the transition from hospital to home. In addition, future implementation studies with larger and more diverse samples of healthcare providers may provide a more comprehensive understanding of factors that influence program adoption, sustainability, and integration into routine clinical care. Multi-site implementation efforts across different NICU settings may further strengthen understanding of how institutional context, resources, and interdisciplinary collaboration shape the successful delivery of family-centered interventions.

## 7. Conclusions

Our findings enhanced the value of designing and implementing therapeutic playgroups for NICU families to facilitate caregiver–infant attachment and outcomes. The main key drivers we identified in this setting include:

- Embedding services into existing workflows (compatibility and appropriateness)
- Offering flexible delivery models (adaptability, feasibility)
- Engaging providers and community partners (readiness and adoption)
- Developing trauma-informed, culturally responsive content (acceptability)
- Addressing caregiver variability through tailored outreach (individual characteristics and engagement)

Our findings should be explored in broader settings, across multiple providers and sites, to enhance generalizability. Confirming these constructs broadly and then explicitly aligning future implementation efforts with these constructs may more effectively bridge gaps in care and support the mental health of families transitioning from the NICU to the community.

## Figures and Tables

**Table 1:** CFIR Mapping Table.

Theme	CFIR Domain	CFIR Construct
Emotional and Developmental Needs of Families	Intervention Characteristics	Relative Advantage
Isolation and Stress During the Transition Home	Outer Setting	Patient Needs & Resources
Alignment with Existing Services	Inner Setting	Compatibility
Feasibility and Integration into Local Programs	Inner Setting/Outer Setting	Networks & Communication/Cosmopolitanism
Caregiver-Related Barriers	Characteristics of Individuals	Self-Efficacy/Individual Stage of Change
Psychologist-Related Barriers	Inner Setting	Available Resources
Organization-Related Barriers	Inner Setting	Available Resources/Access to Knowledge & Information
Integration into Clinical Practice	Inner Setting	Implementation Climate/Access to Knowledge & Information
Delivery Models and Accessibility	Intervention Characteristics/Outer Setting	Adaptability/Patient Needs & Resources
Attendance Incentives	Intervention Characteristics	Cost/Design Quality and Packaging
Provider-Driven Referrals and Buy-In	Process	Engaging—Opinion Leaders/Champions
Direct Caregiver Outreach & Communication	Process/Outer Setting	Engaging—External Change Agents/Executing
Curriculum Development	Intervention Characteristics	Intervention Source/Evidence Strength & Quality
Multidisciplinary Collaboration	Process/Inner Setting	Engaging—Champions/Planning/Networks & Communication

**Table 2:** Thematic Table.

1. Goodness of Fit				
Theme	Description	Sub-Theme	Frequency	Quotes from NICU Mental Health Providers
Emotional and developmental needs of the families	Captures perspectives on how the intervention aligns with caregiver–infant bonding, parental mental health, and developmental support following NICU discharge.		4 out of 4	These families would benefit from additional support after discharge. This is frequently a very difficult adaptation for them. They used to rely on their infant’s medical team, but they are now absent. It is a highly stressful period. Psychologist 2
Isolation and stress during transition home	Encompasses references to emotional distress, loneliness, and lack of support during the immediate post-discharge period.		3 out of 4	What a needed project! Our families need this type of dyadic-focused support to improve mothers’ mental health and infants’ developmental outcomes. These groups will help families adjust to caring for their baby alone at home. Psychologist 3
Alignment with existing programs	Includes perceptions about how the intervention complements or overlaps with current services, workflows, or care philosophies		3 out of 4	We provide integrative behavioral health services, primarily consultation services, to NICU families at ZSFG and UCSF. Our approach is trauma-focused and informed by an Infant Mental Health lens. These therapeutic playgroups will continue the work we started. Psychologist 1
Feasibility and integration of local programs	Referring to the perceived practicality of implementing the playgroup in collaboration with external community programs or local service networks.		4 out of 4	These interventions could be implemented with local community mental health partners, such as the Homeless Prenatal Program or the Good Samaritan. Psychologist 1
2. Implementation Barriers				
Theme	Description	Sub-Themes	Frequency	Quotes from NICU Mental Health Providers
Caregiver-related	Including logistical (e.g., transportation, scheduling), emotional (e.g., trauma, stigma), and perceptual (e.g., mistrust, self-efficacy) challenges that may hinder caregiver participation.	Caregiver Time Constraints	3 out of 4 2 out of 4 3 out of 4 1 out of 4	Parents with medically complex infants can have difficulty attending therapeutic playgroups. For many different reasons, they have so many medical appointments scheduled, other children to take care off, a lack of childcare for older children…Our families may face limitations when using public transportation, and parking can be very difficult. Psychologist 4 Families with newborns hospitalized in the NICU can develop a problematic relationship with their newborn’s medical team… Coming to these groups can bring feelings of being judged, misunderstood, and mistrusted. The mothers I work with feel such deep guilt for their newborn being in the NICU. They might resist going to the therapeutic playgroup, may be concerned about hearing other parents’ stories… Psychologist 2
		Logistical Barriers Healthcare System Mistrust Mental Health Referral Concerns		
Clinician-related	Covers time constraints, limited availability, and competing professional responsibilities that restrict provider engagement with the program.		1 out of 4	Currently, only two psychologists are working less than 50% [Full-Time Equivalent] on the NICU at ZSFG. We would not have the capacity to facilitate these groups. Psychologist 4
Organizational-related	It encompasses systemic and structural limitations such as a lack of funding, staffing shortages, space constraints, and administrative bottlenecks.		4 out of 4	These groups would need to have funding support outside of the department’s available funds to increase family engagement. Psychologist 3
3. Strategies to Address Common Challenges				
Theme	Description	Sub-themes	Frequency	Quotes from NICU Mental Health Providers
Attendance incentives	Refers to suggestions for encouraging participation through tangible (e.g., food, transport support) or intangible (e.g., social recognition) motivators.		3 out of 4	Creating attendance incentives, such as transportation vouchers, snacks, and participation certificates, can help increase Volunteers’ engagement and motivation. Psychologist 3
Integration into clinical practice	Includes strategies for embedding the playgroup into standard workflows, such as discharge planning, care coordination, or clinical follow-up.	Routine Care Incorporation	2 out of 4	It would improve group engagement if the therapeutic playgroup were mentioned on the discharge paperwork and introduced to parents before their infant’s discharge. Psychologist 2 Collaborating with medical staff at [the] NICU is a critical step toward improving the feasibility and integration of this intervention. Psychologist 1
Delivery models and accessibility	Captures ideas related to program format and modality (e.g., in-person, virtual, hybrid) to increase accessibility and caregiver flexibility.		3 out of 4	These families face several competing commitments and barriers to attending in-person groups. Offering a flexible, caregiver-led approach with an online option presents a more equitable solution to meet their needs. Psychologist 4
4. Engagement Strategies				
Theme	Description	Sub-Themes	Frequency	Quotes from NICU Mental Health Providers
Direct caregiver outreach & communication	Involves using targeted communication strategies and materials (e.g., flyers, emails) and partnerships with trusted community organizations to inform and invite caregivers.	Community Partnerships and Outreach Outreach Materials	1 out of 4 1 out of 4	Establishing partnerships with local organizations could support recruitment efforts and foster ongoing collaboration. Psychologist 1 I would recommend having some outreach materials, such as flyers, to hand out to parents in the NICU. Psychologist 3
Provider-driven referrals and buy-in	Encompasses strategies for engaging healthcare staff to support and refer caregivers, including the identification of internal champions and presenting at team meetings.	Program Champion Engagement Provider Referrals and Trusted Relationships Staff Education and Awareness Sessions	3 out of 4 3 out of 4 4 out of 4	You’ll need a champion to help implement these therapeutic playgroups, someone with a strong reputation and experience in the NICU. Psychologist 2 Pediatricians or provider referrals can be a valuable pathway to connect families with the therapeutic playgroup, especially when coming from a trusted source. Presenting the therapeutic playgroup during NICU staff meetings would help strengthen collaboration between medical providers and group facilitators. Psychologist 1
5. Implementation Next Steps				
Theme	Description	Sub-themes	Frequency	Quotes from NICU Mental Health Providers
Curriculum development	Includes planning and designing the playgroup structure, content, and facilitation strategies to ensure program coherence and quality.		2 out of 4	Working with infants who have a history of NICU hospitalization and their caregivers requires a strong infant mental health and trauma-informed approach. Facilitators should be well-versed in evidence-based interventions like Acceptance and Commitment Therapy, Trauma Focus-Cognitive Behavioral Therapy, and dyadic psychotherapy. Psychologist 1
Multidisciplinary collaboration	Refers to involving various stakeholders—such as NICU providers, mental health professionals, and community partners—in program design and rollout.		4 out of 4	In the long run, implementing these therapeutic playgroups for our NICU families will require the development of interdisciplinary collaborations across the NICU, pediatrics, and women’s health. Psychologist 4
Caregivers-related barriers	Covers planning for variability in caregiver readiness, trauma history, and engagement capacity when preparing for future implementation.		4 out of 4	Having multiple sources of funding could help make this project more sustainable in the long term. Psychologist 3

**Table 3:** Mapping Study Findings to CFIR Domains.

CFIR Domain	Study-Mapped Findings
Intervention Characteristics	Strong perceived “goodness of fit” with families’ emotional and developmental needs; value placed on trauma-informed, culturally tailored, and dyadic models (e.g., Child–Parent Psychotherapy, Trauma Focus-Cognitive Behavior Therapy); flexibility and adaptability of delivery methods (in-person, virtual, hybrid).
Outer Setting	Engage community-based organizations for outreach and referrals (e.g., Homeless Prenatal Program)—External barriers: transportation, stigma surrounding mental health—Align with caregivers’ real-world challenges post-discharge
Inner Setting	Organizational barriers: insufficient staffing, limited space, constrained funding; variability in leadership support; partial alignment with clinical workflows, with suggested integration into discharge planning.
Characteristics of Individuals	Clinicians’ workload and limited availability for group facilitation—Staff belief in the value of the intervention—Caregiver stigma regarding mental health, low emotional readiness, and variability in post-discharge capacity to engage
Process (of Implementation)	Importance of identifying internal champions to drive implementation—Strategies: NICU staff meetings, flyers, direct caregiver outreach—Emphasis on timing and method of parent engagement (e.g., during NICU stay vs. post-discharge follow-up)

## Data Availability

All data synthesized in this manuscript will be made available upon publication.
